# eSyM: An Electronic Health Record–Integrated Patient-Reported Outcomes–Based Cancer Symptom Management Program Used by Six Diverse Health Systems

**DOI:** 10.1200/CCI.21.00137

**Published:** 2022-01-05

**Authors:** Michael J. Hassett, Christine Cronin, Terrence C. Tsou, Jason Wedge, Jessica Bian, Don S. Dizon, Hannah Hazard-Jenkins, Raymond U. Osarogiagbon, Sandra Wong, Ethan Basch, Toby Austin, Nadine McCleary, Deborah Schrag

**Affiliations:** ^1^ Dana-Farber Cancer Institute/Brigham and Women's Hospital, Boston, MA; ^2^ Johns Hopkins University School of Medicine, Baltimore, MD; ^3^ Epic, Verona, WI; ^4^ Maine Medical Center, Portland, ME; ^5^ Lifespan Cancer Institute and Brown University, Providence, RI; ^6^ West Virginia University Cancer Center, Morgantown, WV; ^7^ Baptist Medical Center, Memphis, TN; ^8^ Dartmouth Hitchcock Medical Center, Lebanon, NH; ^9^ Lineberger Cancer Center, University of North Carolina, Chapel Hill, NC; ^10^ Memorial Sloan Kettering Cancer Center, New York, NY

## Abstract

Collecting patient-reported outcomes (PROs) can improve symptom control and quality of life, enhance doctor-patient communication, and reduce acute care needs for patients with cancer. Digital solutions facilitate PRO collection, but without robust electronic health record (EHR) integration, effective deployment can be hampered by low patient and clinician engagement and high development and deployment costs. The important components of digital PRO platforms have been defined, but procedures for implementing integrated solutions are not readily available.

**METHODS:**

As part of the NCI's IMPACT consortium, six health care systems partnered with Epic to develop an EHR-integrated, PRO-based electronic symptom management program (eSyM) to optimize postoperative recovery and well-being during chemotherapy. The agile development process incorporated user-centered design principles that required engagement from patients, clinicians, and health care systems. Whenever possible, the system used validated content from the public domain and took advantage of existing EHR capabilities to automate processes.

**RESULTS:**

eSyM includes symptom surveys on the basis of the PRO-Common Terminology Criteria for Adverse Events (PRO-CTCAE) plus two global wellness questions; reminders and symptom self-management tip sheets for patients; alerts and symptom reports for clinicians; and population management dashboards. EHR dependencies include a secure Health Insurance Portability and Accountability Act-compliant patient portal; diagnosis, procedure and chemotherapy treatment plan data; registries that identify and track target populations; and the ability to create reminders, alerts, reports, dashboards, and charting shortcuts.

**CONCLUSION:**

eSyM incorporates validated content and leverages existing EHR capabilities. Build challenges include the innate technical limitations of the EHR, the constrained availability of site technical resources, and sites' heterogenous EHR configurations and policies. Integration of PRO-based symptom management programs into the EHR could help overcome adoption barriers, consolidate clinical workflows, and foster scalability and sustainability. We intend to make eSyM available to all Epic users.

## INTRODUCTION

Among people with cancer, poor symptom control decreases quality of life, increases the need for emergency care,^1-3^ and deters patients from receiving life-prolonging therapy.^4,5^ For recipients of cancer-directed therapy, suboptimal management of chemotherapy side effects and postoperative symptoms leads to distress, delays recovery, and interferes with timely receipt of comprehensive therapy.^6-8^ Suboptimal symptom management also leads to costly emergency department (ED) visits and hospital admissions, which may be preventable.^9-11^ Improving symptom management is critical to improving outcomes and reducing costs for patients with cancer.

**CONTEXT**

**Key Objective**
Is it possible to create and deploy an electronic health record (EHR)-integrated patient-reported outcomes (PRO)-based symptom management program as part of routine clinical practice across multiple health systems?
**Knowledge Generated**
The electronic symptom management program, which has been deployed across six diverse health systems, uses standard, validated patient-reported outcomes; reminders and symptom self-management tip sheets for patients; alerts and symptom reports for clinicians; and population management dashboards. This effort benefited from existing EHR capabilities (eg, a secure Health Insurance Portability and Accountability Act-compliant patient portal), but it has faced technical challenges (eg, the innate technical limitations and heterogenous configurations of EHR systems) and operational hurdles (eg, limited availability of site technical personnel).
**Relevance**
A symptom management program that is designed to optimize postoperative recovery and well-being during chemotherapy, uses validated content, and is embedded within an existing EHR could help overcome adoption barriers, consolidate clinical workflows, and foster scalability and sustainability.

Historically, the cancer care delivery system has used a reactive symptom management strategy making it ill-equipped to anticipate, monitor, and address symptoms before they escalate.^12^ Severe symptoms often receive attention only after causing distress and requiring costly interventions; and mild-moderate symptoms can be left unattended even when early interventions could improve quality of life and prevent the development of severe symptoms. Recent studies suggest that patient-reported outcomes (PROs) help address these shortcomings by enhancing symptom detection and improving symptom control.^9,13^ Studies have found that using telehealth and web-based services to collect electronic PROs (ePROs) from patients with cancer is feasible^14^ and deemed useful by patients and clinicians.^15^ Moreover, collecting ePROs has helped decrease symptom burden, improve quality of life, reduce acute care utilization,^16^ and prolong survival.^13,17^

Efforts to deploy PRO-based symptom management solutions, although limited, have shown promise. However, important questions regarding PRO use in the routine cancer care setting remain since past work has focused on the collection and interpretability of PROs in clinical trials,^18-21^ not every-day practice. First, prior efforts were conducted at large, well-resourced cancer centers.^9,13^ Can these tools have an impact across community and rural settings? Second, prior efforts targeted a limited number of cancer types and symptoms.^9,13,19-22^ Can a common solution be applied across a broader range of cancers, symptoms, and treatments? Finally, prior efforts typically relied on free-standing ePRO applications that may be costly, complicated to deploy, and lack electronic health record (EHR) integration.^23^ Can EHR-integrated solutions offer practical advantages while retaining the same impact on outcomes as free-standing applications?

**FIG 1.**
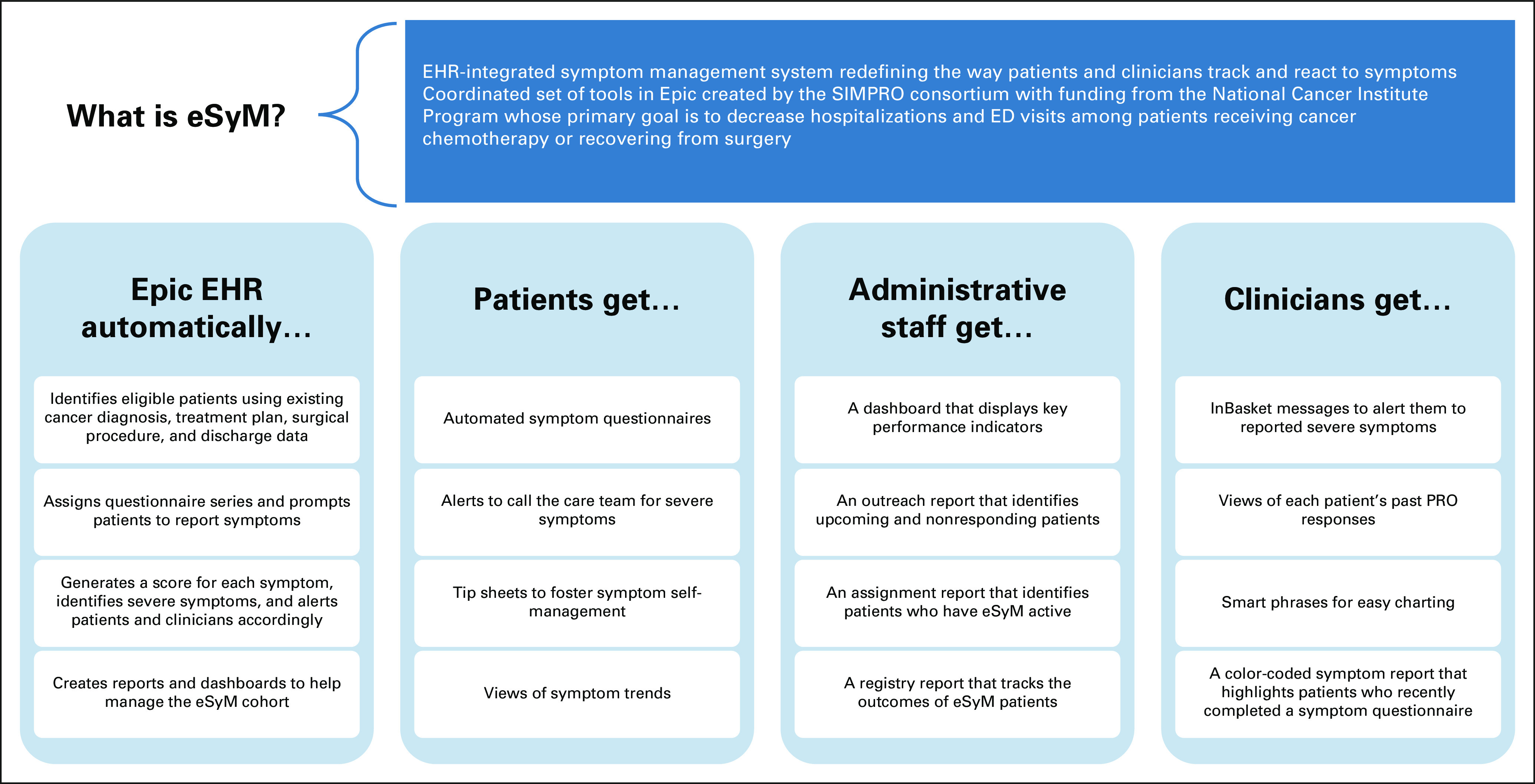
Overview of eSyM. ED, emergency department; EHR, electronic health record; eSyM, electronic symptom management; PRO, patient-reported outcome; SIMPRO, Symptom Management Implementation of Patient-Reported Outcomes in Oncology.

Recognizing this opportunity, the National Cancer Institute (NCI) Cancer Moonshot Initiative sponsored the IMPACT (Improving the Management of symPtoms during And following Cancer Treatment) Consortium to improve symptom control for patients with cancer. IMPACT research sites engage patients in systematic symptom reporting and guideline-based clinical management, as well as evaluate the effects on patient-centered outcomes.^24^ IMPACT funded three research projects to address gaps in symptom management, including the Symptom Management Implementation of Patient-Reported Outcomes in Oncology (SIMPRO) Consortium. The SIMPRO consortium sought to develop, implement, and evaluate the impact of a multicomponent electronic symptom management system (eSyM) for patients recovering from surgery or receiving chemotherapy (Fig 1). To create a scalable, cost-effective, and practicable solution, experts in surgery, medical oncology, symptom management, nursing, and informatics sought to build an EHR-integrated system with sufficient flexibility to be used across multiple health care systems and by various patient populations. This manuscript describes the development of eSyM, including its key components, workflows, and capabilities.

## METHODS

### Setting and Participants

The SIMPRO consortium is composed of six US cancer centers (Data Supplement) that were chosen because they had minimal pre-existing ePRO experience; were Epic (Verona, WI) EHR users; and provided care to diverse patient populations including rural, elderly, and vulnerable groups who might benefit from an ePRO solution. The SIMPRO consortium also includes Epic as a key partner. Epic builds and maintains health record software for 250 million people worldwide, including 73% of the NCI's Designated Cancer Centers.^25,26^ Collaboration between the cancer centers and Epic was crucial, since the primary goal was to develop an EHR-integrated system with the potential to reach the broader oncology community.

### Team and Process

The SIMPRO team (Data Supplement) included three representatives from each SIMPRO site: a principal investigator physician, an Epic support technical lead, and a project manager. Epic was represented by staff from the Beacon (oncology), Healthy Planet (registry and reporting), and MyChart (patient portal and questionnaires) teams. Subject matter experts in questionnaire design, cancer care delivery, and implementation science were engaged as needed.

The development process was agile and collaborative.^27^ It incorporated user-centered design principles that required robust engagement from patients, clinicians, and health system administrators. The build team met weekly to review design components, troubleshoot technical issues, and develop the prototype system; site investigators and technical leads met monthly to review build requests and progress; and content experts and patients participated as needed. Design and configuration decisions, which were primarily the responsibility of the build team, were guided by three principles—create a system that could reduce hospitalizations and ED visits, was easy to use, and could be implemented at any Epic-based health care institution. The consensus-based decision-making process required input and approval from all sites to ensure the final solution was practically robust and widely supported.

The program implementation schedule followed a randomized stepped-wedge design with six go-live waves, each lasting 6 months. During each go-live wave, one site introduced medical oncology eSyM and another site introduced surgery eSyM. The medical oncology and surgery go-lives for each site were staggered to occur during different waves to permit a rigorous evaluation of eSyM's impact. Technical preparation, build, and testing typically require approximately 3 months of work before launch; program validation and optimization require approximately 1 month after launch; and the effort required has been relatively consistent across sites. Postlaunch eSyM updates have been made if they foster the system's primary goal and are endorsed by all SIMPRO sites. This manuscript describes eSyM features that are common to all six sites and identifies aspects that required site-specific configuration.

### Specifications

Several program requirements were established a priori, on the basis of findings from previously published studies, the team's prior experiences, and the overarching program goal:

1. We created two independent programs—one for medical oncology and one for surgery—that could function in concert. Although eSyM functionality could vary on the basis of whether a patient had surgery or was receiving chemotherapy, it would not vary on the basis of cancer type, chemotherapy type, or surgery type, because it would be easier for health systems to deploy a standardized versus a customized system.
2. The initial phase of the project focuses on patients undergoing surgery or receiving chemotherapy for gynecologic, thoracic, and GI malignancies. This eased the burden of deployment and allowed for comparison to patient populations for which eSyM was not launched. Also, eSyM was created for English-speaking adults; future iterations will address other cancer types, languages, and ages.
3. eSyM focuses on detecting and managing symptoms between clinic visits using the extensively validated Patient-Reported Outcome-Common Terminology Criteria for Adverse Events (PRO-CTCAE) instrument.^28,29^ Pictogram questions for overall well-being and functional status were added to contextualize symptom responses.
4. All content, including questionnaires and patient self-management tip sheets, is available from or shared with the public domain. eSyM relies on existing Epic software capabilities, with a priority placed on automating processes whenever possible, to reduce barriers to adoption.

## RESULTS

The eSyM program was designed to help patients, clinicians, and staff work together to reduce hospitalizations and ED visits during chemotherapy and following surgery (Fig 1). eSyM has two core technical features, the patient registries and questionnaire series, and multiple supporting tools.

### Core Features

Two patient registries automatically identify eligible patients. The medical oncology registry identifies patients who have a new treatment plan in the Beacon oncology module scheduled to begin in the next 30 days, a relevant International Classification of Diseases-10 cancer diagnosis code, and an encounter at a participating site. The surgery registry identifies patients who have a relevant current procedural terminology code in the OpTime module, a surgery date in the next 30 days, and a recent encounter with a participating surgical department or surgeon. The surgery registry focuses on cancer-directed procedures, excluding diagnostic interventions (eg, biopsies), but does not require a cancer diagnosis, because this information may not be available until the pathology report is finalized several weeks after surgery. Groupers define relevant cancer diagnoses or surgeries. They can be updated as the program evolves or customized by sites if they decide to modify the program.

**FIG 2.**
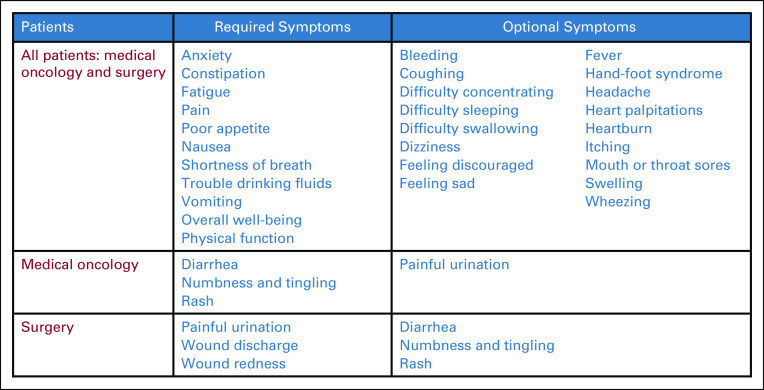
eSyM questionnaire items. eSyM questionnaires includes 12 required symptom items, two required general context items, and up to 20 optional symptom questions. Symptom questions are based on the PRO-CTCAE, which asks one-three questions per symptom and aggregates responses into a four-point scale (0 = no symptoms, to 3 = severe symptoms). The complete PRO-CTCAE questionnaire system and terms of use can be accessed at the National Cancer Institute.^30^ eSyM, electronic symptom management; PRO-CTCAE, Patient-Reported Outcome-Common Terminology Criteria for Adverse Events.

Two eSyM questionnaires were developed—one for medical oncology and one for surgery (Fig 2). Nine symptoms and two pictogram questions are common to both questionnaires; three symptoms are unique to each questionnaire. Also, patients can report up to 20 additional symptoms at their discretion. Symptom reports are gathered using the validated PRO-CTCAE instrument.^31^ This tool asks about a symptom's frequency, severity, and/or interference; and uses an algorithm to translate responses into a four-item score (0 = no, 1 = mild, 2 = moderate, and 3 = severe).^28,29^ Pictogram questions assess overall well-being and physical function to help contextualize symptom responses (Data Supplement).^32,33^

The medical oncology questionnaire series becomes active (ie, the patient can start responding) the day after the first treatment is completed. A questionnaire can be completed up to twice/week until 180 days after the treatment plan is initiated or the series is discontinued, whichever comes first. The surgery questionnaire series becomes active the day after a patient is discharged from the hospitalization during which the index procedure was performed. A questionnaire can be completed up to three times/week for weeks 1-2, up to twice/week for weeks 3-4, and up to once/week for weeks 5-8. The schedules were chosen to focus on times when patients are most likely to experience severe symptoms. Questionnaires take < 5 minutes. Responses file automatically to the patient's medical record.

To avoid confusion and streamline workflows, patients are only asked to complete one questionnaire series at a time. If an active eSyM patient begins a new chemotherapy plan or has another surgery, then the old questionnaire series ends and a new series starts. There is no limit to the number of times a questionnaire series can be applied. For patients who opt out of eSyM, program staff can manually discontinue questionnaire series.

**FIG 3.**
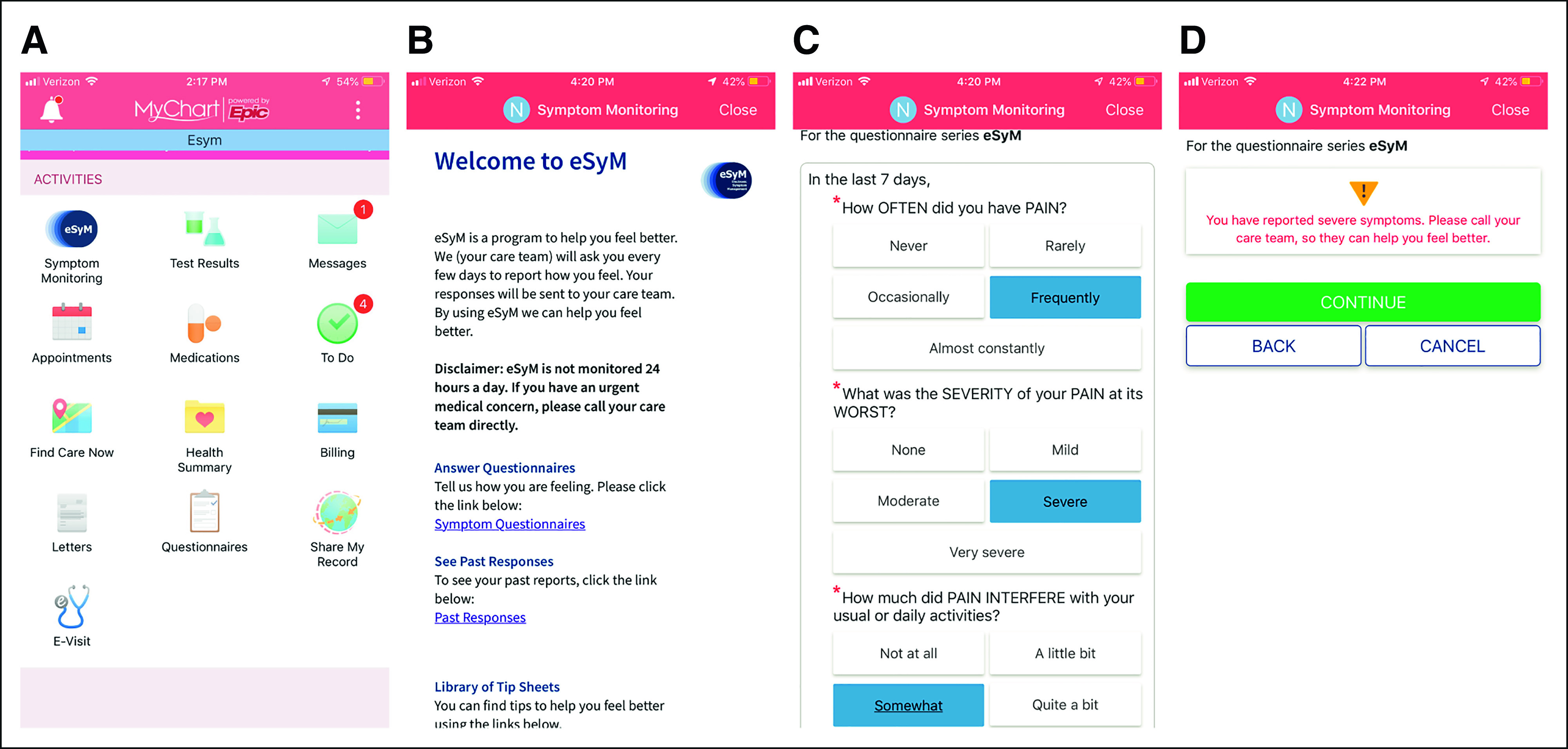
Patient-facing eSyM tools. Four views of the smartphone version of MyChart, Epic's patient-facing mobile application (*from left to right*). (A) The MyChart mobile home screen with a link to the eSyM program. (B) The eSyM Welcome Page, where the user can answer questionnaires, view past responses, and access tip sheets. (C) The eSyM questionnaire for pain, where the patient reports the severity, frequency, and interference of selected symptoms. (D) The eSyM Patient Alert Screen, which is presented to patients who have reported severe symptoms. eSyM, electronic symptom management.

Patients access all eSyM tools through the MyChart portal (Fig 3). An eSyM homepage provides (1) background information outlining the purpose of the program and presenting a disclaimer that it is not monitored 24-7; (2) a link to respond to the current questionnaire; (3) a view of previously reported symptoms; and (4) a library of self-management tip sheets. Tip sheets were developed to empower patients to manage symptoms at home (Data Supplement). Each one includes things patients can do (1) on their own, (2) with over-the-counter medications, and (3) with the help of the care team, as well as guidance for when to call the care team. Tip sheets were developed de novo by SIMPRO Consortium clinicians to avoid copywrite encumbrances from previously published standards and to ensure identical resources were available to all eSyM patients. All eSyM tools are equally accessible via the mobile and web versions of MyChart.

### Supporting Tools

The eSyM program generates three context-dependent patient messages: (1) for new patients, a welcome message with program instructions; (2) for patients with unanswered questionnaires, a reminder message to complete the questionnaire with a direct link; and (3) for patients who report severe (ie, grade 3) symptoms, a message encouraging the patient to contact the care team. All patient messages are delivered via MyChart. If permitted by the health care system, patients can choose to have reminders delivered via e-mail or text.

Multiple tools are available to support clinicians caring for eSyM patients. InBasket messages alert the care team when patients submit questionnaires with severe symptoms (Data Supplement). Alerts are monitored during business hours only; follow-up calls are conducted as needed on the basis of clinical judgment. Reported symptoms and symptom scores, including trends, appear in the EHR in multiple locations (Data Supplement). Clinicians use ‘smart phrases’ to add symptom scores to notes to aid documentation. Flags can indicate which patients on a clinic schedule are part of the eSyM program.

**FIG 4.**
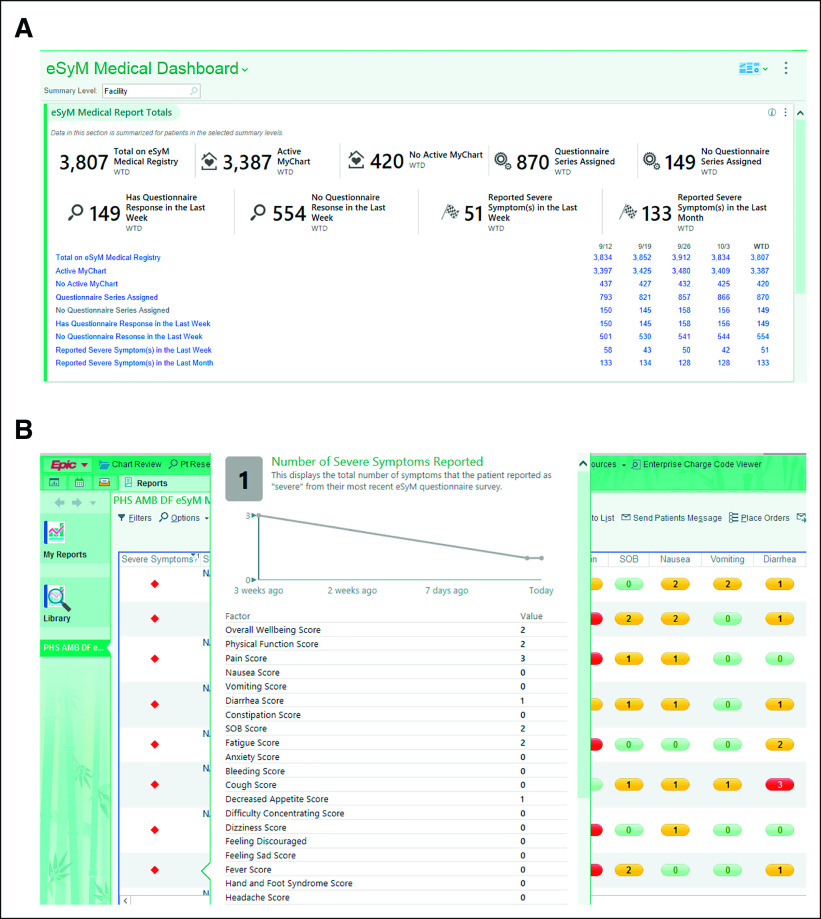
Key clinician-facing eSyM tools. (A) The eSyM dashboard shows key performance indicators and can be used to access reporting workbench reports that facilitate population management. It can be customized by each user to focus on a clinician, department, or service area. (B) The Symptom Management Report shows all submitted questionnaires from the past week. It is color-coded to highlight patients reporting severe symptoms and includes tool-tips that show symptom trends. It can be filtered and sorted on the basis of multiple features including symptom severity. Additional symptom scores and trends are displayed by hovering over the severe symptom indicator column. eSyM, electronic symptom management.

A dashboard and four workbench reports are used to manage the active eSyM cohort (Fig 4A). The measures section displays nine real-time key performance measures. The outcomes section displays the number of ED visits, urgent care visits, and hospitalizations per 1,000 patients. The reports section provides links to four reporting workbench reports that deliver detailed information about eSyM patients. The All Symptoms Weekly Report identifies patients who completed an eSyM questionnaire in the last 7 days (Fig 4B). It highlights the total number of moderate (grade 2) and severe (grade 3) symptoms, trends in these values, and the most recent scores for six high-priority symptoms. The Outreach Report identifies patients who are about to have eSyM assigned or have had eSyM assigned but have not responded in the last 2 weeks. The All Registry Patients report helps track active eSyM patients; and the All Assigned Patients report records outcome events. Users can customize the dashboard and reports to fit specific needs (eg, focus on a specific department or provider).

### Current Status

Four of six planned go-live waves have been completed on schedule. As of August 2021, two sites are live with eSyM medical oncology, two sites are live with eSyM surgery, and two sites are live with both. After the last two go-live waves, all six sites will be live with both surgery and medical oncology (estimated Spring 2022).

**FIG 5.**
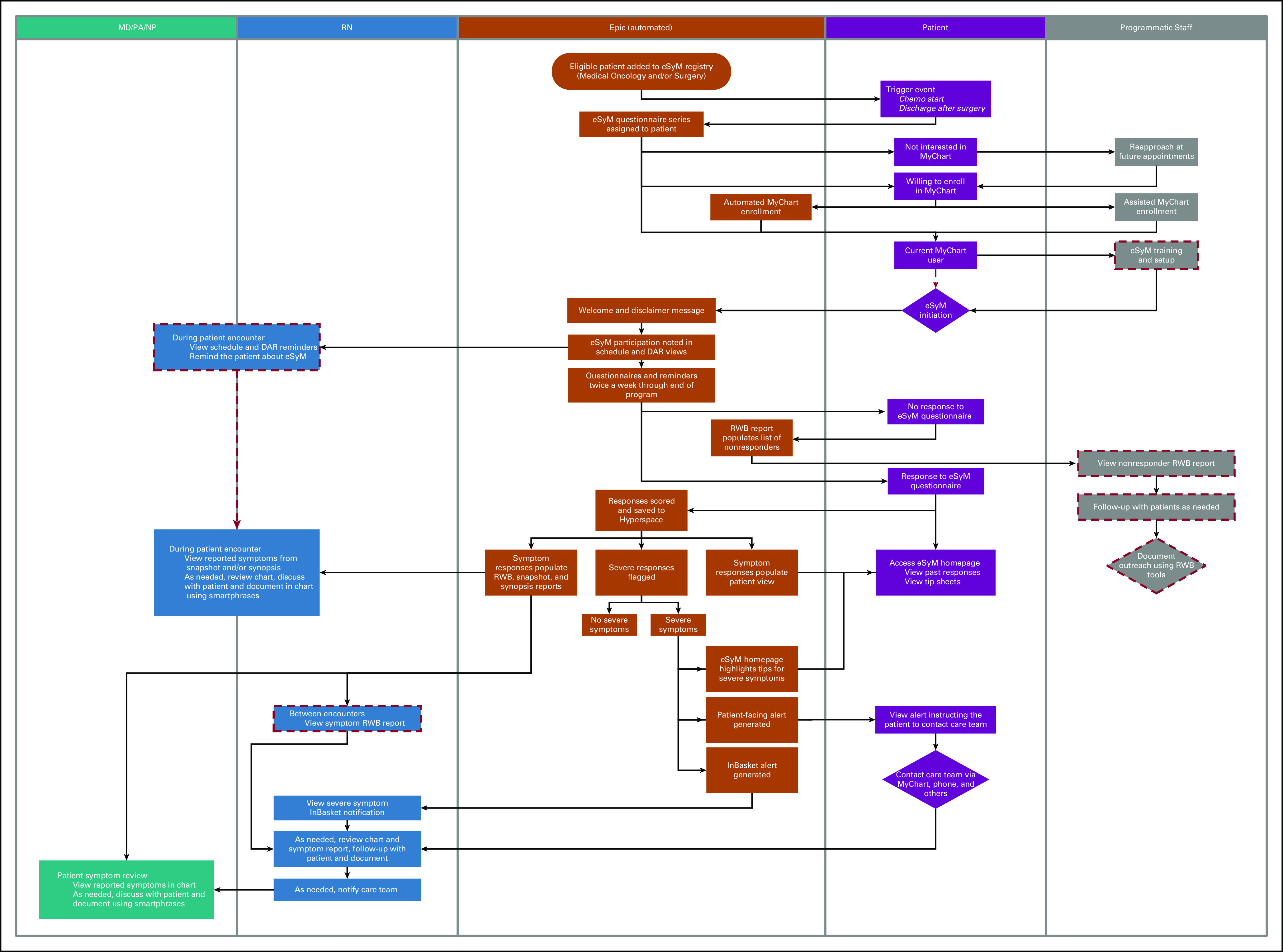
Process flow for eSyM. Automated (Epic-based) functionality occupies the center swim lane, with adjacent swim lanes indicating tasks accomplished by the patients, the program nurses who provide supportive care, the MD, NP, and PA staff who oversee care, and the programmatic staff who support the eSyM program. Medical oncology patients are considered active on eSyM until their chemotherapy treatment plan is discontinued or 180 days later, whichever comes first. Surgery patients are considered active on eSyM for 60 days. eSyM assignments can be manually removed at any time by study staff. Red dashed lines indicate processes that may be site specified (ie, may only be used on some sites). DAR, department appointment report; eSyM, electronic symptom management; MD, medical doctor; NP, nurse practitioner; PA, physician assistant; RN, registered nurse; RWB, reporting workbench.

Since September 2019, 3,352 unique patients have submitted 26,268 symptom questionnaires. Across all sites, two third of patients with patient portal access completed at least one eSyM questionnaire and one third completed eSyM multiple questionnaires. Patient feedback has been positive; reasons for not using the program include (1) limited access to internet-enabled devices, (2) limited technology confidence, (3) no symptoms to report, and (4) no encouragement from the care team to submit symptom reports. In response to this feedback, additional training materials outlining the importance of PRO collection have been created, and alternative ePRO collection methods, including iPads in clinic and proxy reporting at home, have been offered. A process-flow diagram (Fig 5) summarizes the interplay between eSyM's core features and its primary users. Some sites have already expanded deployment beyond the original gynecologic, thoracic, and GI cancers; and some sites have extended eSyM to non–English-speaking patients. The long-term plan is to build eSyM into Epic's Foundation System.

## DISCUSSION

The SIMPRO consortium created eSyM to reduce hospitalizations and ED visits and improve quality of life among patients with cancer. The program includes tools that address the needs of three distinct user groups. Patients can complete symptom questionnaires, view past responses, receive guidance on severe symptoms, and access self-management tip sheets. Clinicians can view symptom trends, receive severe symptom alerts, and document interventions. Program staff can use population health management tools to oversee and manage the eSyM cohort. The eSyM program relies on multiple pre-existing Epic capabilities, including registries, reports, questionnaires, best practice advisories, and the patient portal. Two years after eSyM build started, the program has been deployed at 6 health systems and used by 3,352 patients.

**FIG 6.**
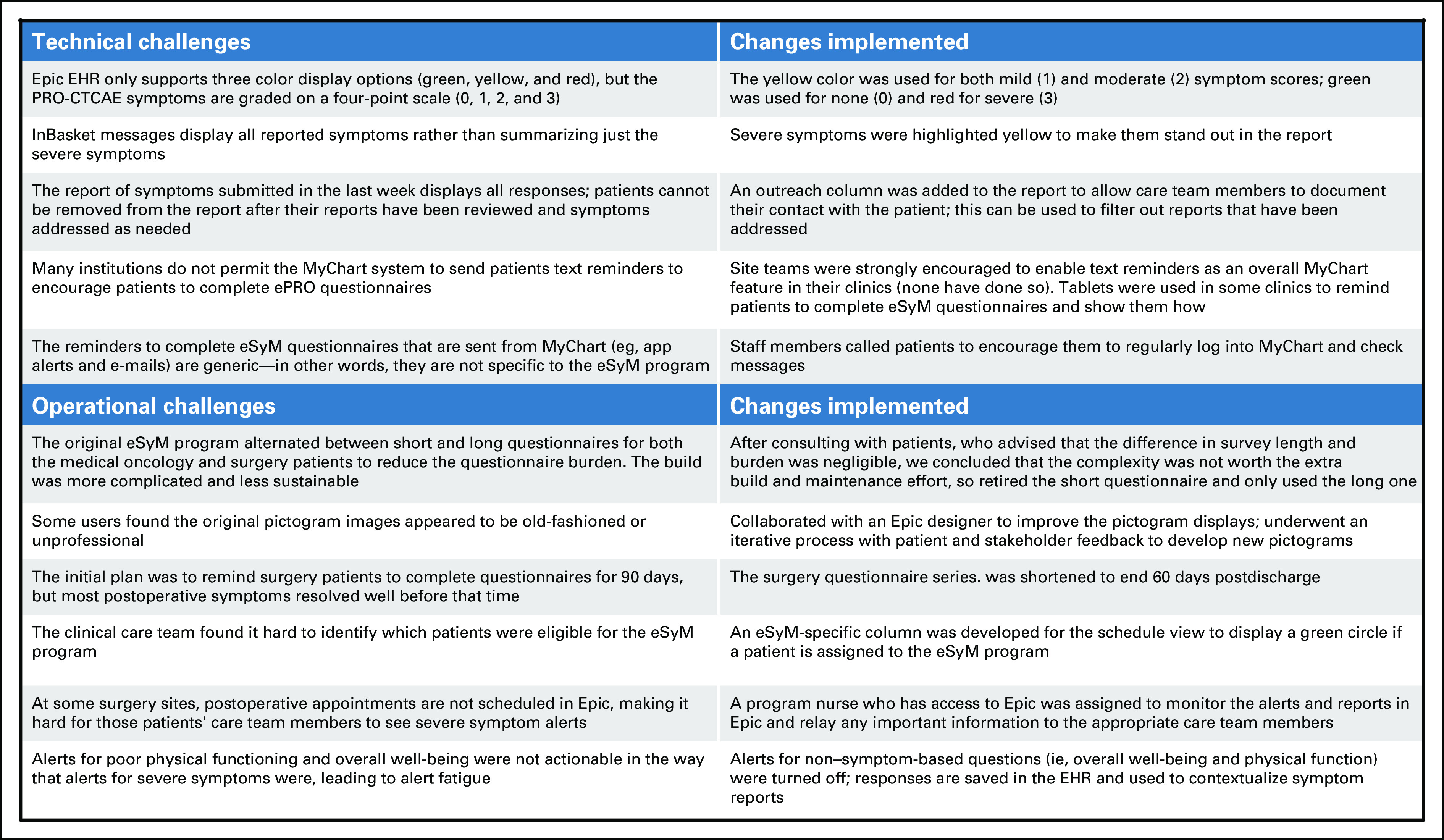
Examples of eSyM challenges and solutions. EHR, electronic health record; ePRO, electronic patient reported outcome; eSyM, electronic symptom management; PRO-CTCAE, Patient Reported Outcome-Common Terminology Criteria for Adverse Events.

Developing and deploying eSyM presented technical and operational challenges (Fig 6). Relying on the existing capabilities of the EHR-system imposed design constraints. For example, the PRO-CTCAE tool assigns a score to each symptom using a four-point scale, but the display options in the reporting module only offered three colors. So, both grade 1 and 2 symptoms appear yellow, with grade 0 and 3 symptoms appearing green and red, respectively. There was a constant need to balance the desire to have eSyM be consistent across all sites with the reality that workflows and Epic configurations vary by site. Although most of eSyM is consistent across sites, variable configuration was required for InBasket routing, report displays, and some registry inclusion criteria. Even with substantial assistance from Epic, aspects of testing, approval, and migration had to be carried out by each site's technical team. These teams had competing demands, notably including those related to the COVID-19 pandemic. Some clinicians expressed concern that eSyM could add to their workload. Considering that burnout is a major concern,^34,35^ we plan to assess the impact of eSyM on clinician workload. Initial observations indicate that clinician impact has been modest and the eSyM program can be easily integrated into existing workflows. The volume of severe alerts has been manageable for clinics. That said, efforts to bolster nurse and physician use of the population management tools and individual patient symptom summary reports are needed. Although baseline patient portal enrollment was high (ie, > 50%) across sites, achieving high levels of patient participation in eSyM has required direct encouragement from the care team. On the administrative side, dedicated support from a program coordinator at each site has been crucial to enable program trainings, patient outreach, and system validation. Some sites, however, have faced difficulties integrating this new coordinator role into their pre-existing workflows. These challenges, which have been highlighted by others,^36^ have presented learning opportunities that have helped prepare for subsequent go-lives. They also represent ongoing barriers to scaling eSyM across diverse, real-world health care systems.

Creation of an ePRO-based symptom management program that is fully integrated into the EHR represents a milestone in the evolution of PROs as a tool to support routine cancer care delivery. As evidence demonstrating the utility and effectiveness of ePRO-based solutions grow, health systems may wonder if they should deploy free-standing versus EHR-integrated solutions. Compared with freestanding, an integrated solution provides efficiencies for patients, clinicians, operations, and technical staff, but it also imposes constraints and limitations (Data Supplement). Considering that health care systems sometimes lack the time and resources needed to support multiple clinician- and/or patient-facing applications, an option that can be disseminated via a major EHR vendor may offer distinct advantages.

Future work will include measuring the impact of eSyM on patients' symptom burden and need for acute care, optimizing eSyM functionality and usability, and extending eSyM to other sites and populations (Data Supplement). Quantitative and qualitative data are being collected from patients and key stakeholders to understand facilitators and barriers to eSyM adoption and utilization. Preliminary findings from SIMPRO suggest that limited internet access and low computer literacy remain as significant barriers to the routine use of ePRO-based symptom management solutions in rural and resource-poor areas. These barriers need to be overcome to prevent this new technology from exacerbating existing disparities.
